# PD-1 immunobiology in glomerulonephritis and renal cell carcinoma

**DOI:** 10.1186/s12882-021-02257-6

**Published:** 2021-03-06

**Authors:** Colleen S. Curran, Jeffrey B. Kopp

**Affiliations:** 1 Critical Care Medicine Department, Clinical Center, NIH, BG 10 RM 2C135, 10 Center Drive, Bethesda, MD 20814 USA; 2 Kidney Disease Section, NIDDK, NIH, Bethesda, MD USA

**Keywords:** Vitamin D3, Glutathione, 5’ AMP-activated protein kinase (AMPK)

## Abstract

**Background:**

Programmed cell death protein (PD)-1 receptors and ligands on immune cells and kidney parenchymal cells help maintain immunological homeostasis in the kidney. Dysregulated PD-1:PD-L1 binding interactions occur during the pathogenesis of glomerulopathies and renal cell carcinoma (RCC). The regulation of these molecules in the kidney is important to PD-1/PD-L1 immunotherapies that treat RCC and may induce glomerulopathies as an adverse event.

**Methods:**

The expression and function of PD-1 molecules on immune and kidney parenchymal cells were reviewed in the healthy kidney, PD-1 immunotherapy-induced nephrotoxicity, glomerulopathies and RCC.

**Results:**

PD-1 and/or its ligands are expressed on kidney macrophages, dendritic cells, lymphocytes, and renal proximal tubule epithelial cells. Vitamin D3, glutathione and AMP-activated protein kinase (AMPK) regulate hypoxic cell signals involved in the expression and function of PD-1 molecules. These pathways are altered in kidney disease and are linked to the production of vascular endothelial growth factor, erythropoietin, adiponectin, interleukin (IL)-18, IL-23, and chemokines that bind CXCR3, CXCR4, and/or CXCR7. These factors are differentially produced in glomerulonephritis and RCC and may be important biomarkers in patients that receive PD-1 therapies and/or develop glomerulonephritis as an adverse event

**Conclusion:**

By comparing the functions of the PD-1 axis in glomerulopathies and RCC, we identified similar chemokines involved in the recruitment of immune cells and distinct mediators in T cell differentiation. The expression and function of PD-1 and PD-1 ligands in diseased tissue and particularly on double-negative T cells and parenchymal kidney cells needs continued exploration. The possible regulation of the PD-1 axis by vitamin D3, glutathione and/or AMPK cell signals may be important to kidney disease and the PD-1 immunotherapeutic response.

## Background

The kidneys perform diverse functions in maintaining human health. These include removing metabolic waste products; contributing to water, electrolyte and acid-base homeostasis; controlling blood pressure via regulation extracellular volume and production of renin and angiotensin; regulating vitamin D3 synthesis and metabolism; and producing erythropoietin which is critical for red blood cell production [1]. Shifts in oxygen consumption or plasma vitamin D3 (1α-25(OH)_2_D_3_) levels disrupt various kidney functions [2, 3] and contribute to the functions of the programmed cell death protein (PD)-1 receptor and its ligands (PD-L1, PD-L2) [4, 5]. The expression of these checkpoint molecules on kidney immune cells and epithelial cells [6] is associated with some forms of glomerulonephritis and renal cell carcinoma (RCC) [7, 8]. PD-1 antibodies are being assessed as therapies for renal cell carcinoma (RCC) and other cancers, and in this context, glomerular and tubulointerstitial disease is a prominent immune-related adverse event [9–11].

To explore the nephrotoxicity of PD-1 immunotherapies, we examined the function and expression of PD-1 molecules in kidney homeostasis and disease. Because nephrotic and nephritic glomerulopathies and tubulointerstitial nephritis occur in response to PD-1 immunotherapy, we reviewed the PD-1 axis on immune and parenchymal cells in these disorders. The pro-inflammatory responses in glomerular and tubulointerstitial disease were contrasted with the immune tolerance known to occur in RCC through the expression of PD-1 molecules on immune and kidney parenchymal cells. Possible cell signals in regulating the kidney PD-1 axis were examined with a particular focus on the kidney epithelium. The similarities and differences between these diseases may provide insight into the nephrotoxicity of PD-1 immunotherapies.

### The PD-1 axis in the healthy kidney

Resident innate immune cells in the renal interstitium include macrophages [12], dendritic cells (DCs) [13], and mast cells [14]. Intravenous injection of small immune complexes in a murine model showed that macrophages, localized around peritubular capillaries, recognize immune complexes via Fc receptors (FcRs), such as FcγRIV, and possibly monitor the trans-endothelial transport of albumin into the kidney interstitium [12]. The expression of PD-1 or its ligands, PD-L1 and PD-L2, on these macrophages in healthy tissue has yet to be fully explored. PD-L1 and PD-L2 have been identified on murine interstitial DCs and on DCs in the kidney-draining lymph, which capture low-molecular weight antigens and peptides [15]. Moreover, PD-L1^+^ DCs in kidney lymph nodes promote tolerance via PD-L1 binding interactions with PD-1 expressed on cytotoxic T cells in the draining lymph [16]. Lastly, mast cells localize to blood vessels, epithelial tissues and neural connective tissue (*i.e.* the three layers of connective tissue that surround each nerve) *in vivo* [14] and express PD-L1 and PD-L2 on murine bone marrow-derived mast cells *in vitro* [17]. PD-1 immunotherapies may therefore alter the function of PD-1 and its ligands on various immune cells in the healthy kidney.

**Fig. 1:**
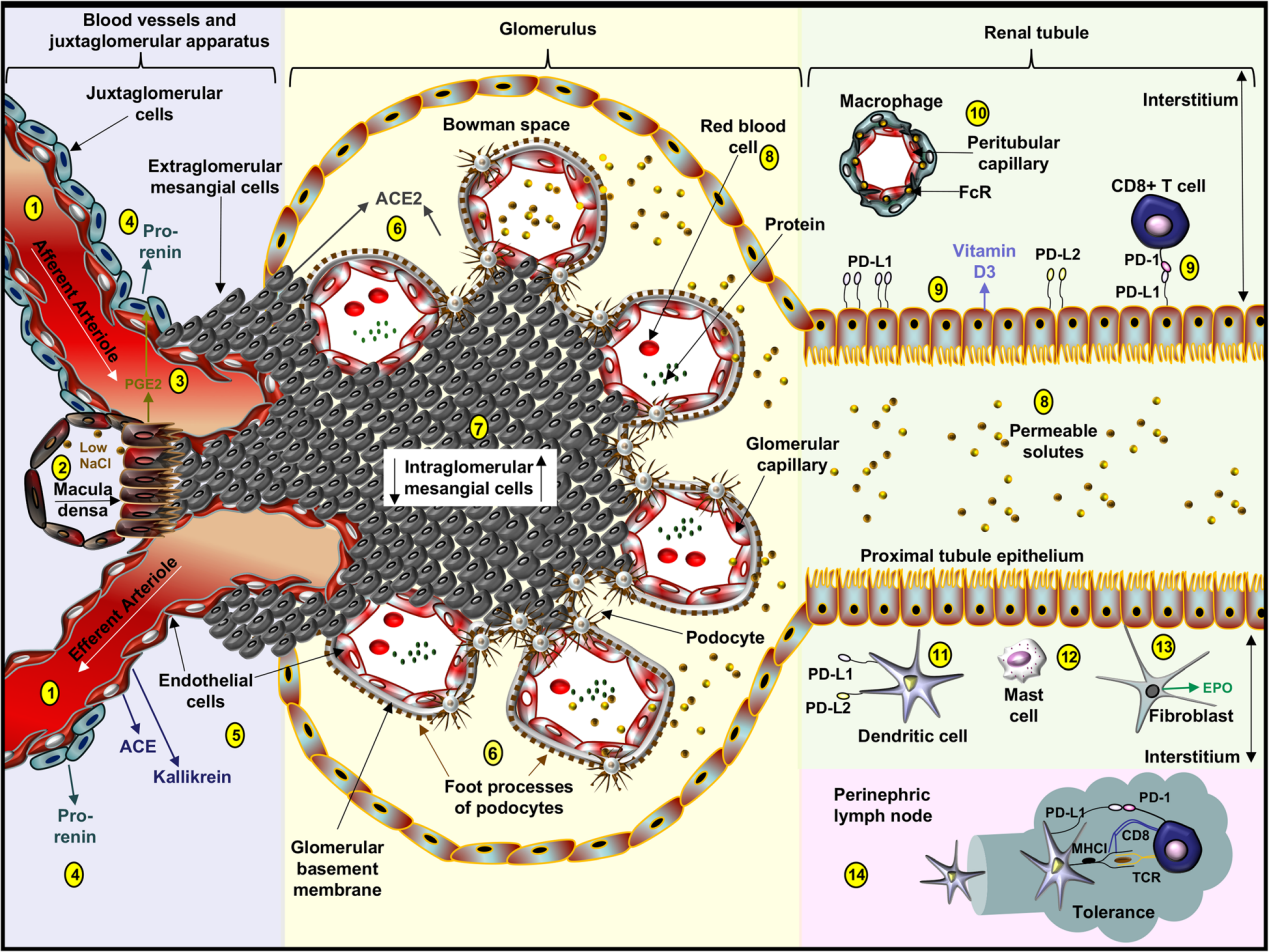
Healthy juxtaglomerular apparatus, glomerulus, renal tubule, and perinephric lymph node. (1) The glomerular capillary network is supplied by the afferent arteriole and drained by the efferent arteriole. (2) Specialized epithelial cells in the macula densa sense NaCl concentrations in the distal tubule. (3) Low NaCl concentrations induce cells in the macula densa to secrete prostaglandins (PGE2), which promote afferent arteriolar dilation. (4) PGE2 stimulates the release of pro-renin from juxtaglomerular cells, predominantly located around the afferent arteriole. (5) Pro-renin is cleaved into renin by endothelial cell kallikrein and both renin and angiotensin converting enzyme (ACE) are key enzymes in the renin-angiotensin system (RAS). (6) A trilaminar structure in the glomerular capillary wall, composed of the endothelium, glomerular basement membrane, and podocyte foot processes, provides a size-selective and charge-selective filter regulating passage of macromolecules from plasma into the urinary space. Podocytes can produce ACE2. (7) Extraglomerular and intraglomerular cells provide structural support and can produce cytokines and ACE2. (8) Impermeable proteins and blood cells remain in the capillaries but permeable solutes (*e.g.,* NaCl, glucose), small molecules, and many proteins are filtered into Bowman space. (9) Proximal tubule epithelial cells produce vitamin D3 and express PD-L1 and PD-L2 where PD-L1 may be integral in CD8 T cell tolerance. (10) Macrophages expressing Fc receptors surround peritubular capillaries and regulate trans-endothelial transport of molecules into the renal interstitium. (11) Interstitial dendritic cells express PD-L1 and PD-L2. (12) Mast cells are infrequently identified in the healthy interstitium. (13) Fibroblasts generate erythropoietin (EPO). (14) Dendritic cells regulate tolerance by presenting innocuous peptides to CD8^+^ T cells in the renal lymph in association with PD-L1 ligation to CD8 PD-1

Human primary renal proximal tubular epithelial cell PD-L1 and PD-L2 expression has been shown *in-vivo* and *in vitro*. In biopsies of patients with renal allografts, PD-L1 and PD-L2 mRNA are upregulated and surface expression of PD-L1 was present on infiltrating cells and epithelial cells in the tubulointerstitium. Moreover, *in vitro* PD-L1 blockade of tubular epithelial PD-L1 binding interactions with PD-1 on CD4^+^ and CD8^+^ T cells reduces alloreactive T cell proliferation and cytokine production [6], suggesting a protective effect of PD-L1 on the tubular epithelium (Fig. 1). Although PD-L1 is expressed on fibroblasts and endothelial cells in extra-renal tissues, the presence of this checkpoint molecule on these cells in the kidney has not yet been assessed [18]. The expression of major histocompatibility class (MHC)-II molecules on podocytes and mesangial cells [19, 20] suggests that checkpoint molecules may be expressed on these cells, as is the case with professional antigen presenting cells (*e.g.* macrophages, DCs, and B cells) (Fig. 1). Because PD-1 ligands appear to promote tissue homeostasis, continued research into the function of these molecules on kidney parenchymal cells in response to disease and PD-1 immunotherapies appears warranted.

### The PD-1 axis in glomerulopathies and tubulopathies

Glomerulopathies are acute or more often chronic kidney disorders that develop in the glomeruli, although associated tubulointerstitial injury is common [21]. Glomerulopathies include both non-inflammatory (nephrotic) and inflammatory forms (nephritic) and may evolve strictly in the kidney (primary) or in response to systemic disease (secondary) [22]. Both nephrotic and nephritic syndromes have been associated with anti-PD-1 therapy (Table 1). The pathophysiology of the glomerulopathies is diverse. Identified factors may include viral infections (*e.g.*, cytomegalovirus, Epstein-Barr virus, hepatitis C virus, herpes simplex virus), autoimmune disorders (*e.g.*, systemic lupus erythematosus (lupus), Goodpasture syndrome, polyarteritis), certain drugs (*e.g*., antibiotics, diuretics, chemotherapeutics) [21, 34–36], genetic mutations (*e.g*., (*Apolipoprotein L1* (*APOLI*)), and unknown factors associated with idiopathic disease (*e.g.,* minimal change disease (MCD)). The development of glomerulopathies secondary to PD-1 immunotherapy is an increasingly recognized complexity of unknown etiology. Patients administered proton pump inhibitors, cytotoxic T-lymphocyte-associated protein 4 (CTLA-4) antibodies, or nonsteroidal anti-inflammatory drugs (NSAIDs) may have a greater risk to renal toxicities in response to PD-1 immunotherapy, resulting in fatal toxic events in as little as 2 weeks compared to greater than a month with PD-1 immunotherapy alone [37, 38]. Understanding the functions of PD-1 receptors in the healthy kidney and disease states that provoke tolerogenic or autoimmune responses may be informative to PD-1 nephrotoxicity clinical presentation, treatment, and algorithms for the use of PD-1 immunotherapy after the resolution of this adverse event.

**Table 1:** Glomerulopathy as a complication of anti-PD-1 immunotherapy in 13 cases

Underlying Disease	Age Sex	Disease Treatment	Syndrome	Syndrome Treatment	Result
Metastatic clear cell renal carcinoma [11]	70 M	10 months of nivolumab 3 mg/kg every 2 weeks subsequent to pazopanib 600 mg daily	Diffuse tubular injury with vacuoles and immune complex-mediated glomerulonephritis with cellular crescents and necrosis	Methylprednisolone 40 mg intravenously 2x/day that was increased to 3x/day, 1g/day, and tapered	Discharged
Metastatic squamous cell anal carcinoma [23]	75 F	2 months (5 cycles) of 2.4 mg/kg nivolumab monotherapy subsequent to colostomy with combined 5-fluororuacil and mitomycin C with radiation	Membranoproliferative glomerulonephritis	Prednisone 40 mg daily	Deceased
Papillary renal cell carcinoma type 2 [24]	62 M	4 cycles of nivolumab 3 mg/kg every 2 weeks subsequent to a cMET inhibitor (INC280), everolimus pazopanib	Early FSGS, due to nivolumab or as a paraneoplastic sign, an acute tubular necrosis, or a postrenal obstruction	IV pulses of 1000 mg methyl-prednisolone for 3 days, followed by prednisone 60 mg/day and mycophenolate mofetil 750 mg twice daily	Discharged, relapse, deceased
Metastatic lung adeno-carcinoma [25]	71 F	Pembrolizumab following completion of carboplatin and pemetrexed treatment	Focally crescentic pauci-immune glomerulonephritis	Pulse glucocorticoids followed by high-dose glucocorticoids	Resolution of proteinuria and hematuria
Squamous cell carcinoma and the development of infectious enterocolitis [26]	65 M	Pembrolizumab 200 mg, in six infusions, over 4 months subsequent to radiation and cisplatin	Pauci-immune necrotizing crescentic glomerulonephritis with positive peri-nuclear ANCA and myeloperoxidase (MPO)	Methylprednisolone 1000 mg/day for 3 days, followed by prednisone 60 mg/day tapered to 50 mg/day. The patient also received two doses of 1000 mg rituximab	Decreased proteinuria and hematuria
Stage IV non-small-cell lung cancer [27]	67 M	Nivolumab 3 mg/kg every 2 weeks subsequent to bevacizumab combined with pemetrexed plus cisplatin followed by maintenance pemetrexed infusion. Lansoprazole 15 mg/day was also prescribed.	Acute tubulointerstitial nephritis (ATIN)	Lansoprazole was discontinued and administration of 500 mg intravenous methylprednisolone for 3 days followed by 1 mg/kg/day oral prednisolone	Positive drug induced lymphocyte stimulating test (DLST) for lansoprazole and improved kidney function
Metastatic anal canal non-mutated BRAF melanoma [28]	76 F	3 cycles of nivolumab (3 mg/kg) administered 8 weeks after ipilimumab (4 cycles of 3 mg/kg) discontinuation	Nivolumab-induced acute immune interstitial nephritis	Oral prednisolone at a daily dose of 0.5 mg/kg (40 mg) and nivolumab eventually discontinued	Improved kidney function
Stage IV melanoma *BRAF* wild type [29]	68 M	Single dose of pembrolizumab (2 mg/kg) as first-line therapy	Acute renal failure with nephrotic syndrome due to a minimal change disease related to pembrolizumab	Oral prednisolone at 100 mg/day and diuretics administered. Pembrolizumab discontinued.	Renal function restored. Ipilimumab (3 mg/kg) and nivolumab (1 mg/kg) resulted in a confirmed a deep partial response after 3 doses
Metastatic melanoma and prostate cancer in remission [30]	64 M	5 cycles pembrolizumab 2 mg/kg every 3 weeks	Diffuse active tubulointerstitial nephritis with severe acute tubular cell injury	IV methyl-prednisolone 1 g/day for three days followed by oral prednisone 60 mg/day and immunotherapy discontinued	With improved renal function patient resumed treatment with ipilimumab instead of pembrolizumab
Metastatic acral melanoma [30]	78 F	3 cycles of nivolumab 3 mg/kg [omeprazole was also prescribed]	Diffuse active chronic tubulointerstitial nephritis with acute tubular cell injury	IV methyl-prednisolone 1 g/day for 3 days followed by oral prednisone 60 mg daily and immunotherapy discontinued	With improved renal function patient resumed treatment with three cycles of temozolomide
Stage IIA adeno-carcinoma of the lung [31]	57 M	4 cycles of biweekly treatments with nivolumab subsequent to radiotherapy and repeated courses of cisplatin, pemetrexed, and bevacizumab [rabeprazole was also prescribed]	Nivolumab-induced acute tubulointerstitial nephritis with CD163^+^ M2 macrophage infiltration	Prednisolone (55 mg, daily) treatment was initiated. Nivolumab and rabeprazole were discontinued	Renal function improved
Recurrent gastric cancer and liver metastases [32]	68 F	30 cycles of nivolumab subsequent to S-1 plus cisplatin (first-line), paclitaxel monotherapy (second-line) and irinotecan monotherapy (third-line)	Acute granulomatous tubulointerstitial nephritis associated with PD-L1+ lesions and aggregated CD3^+^ T cells	Nivolumab was discontinued, and the patient was treated with methylprednisolone 1.0 mg/kg (40 mg) daily	Nivolumab was reinstated up to a total of 41 cycles without kidney dysfunction but the cancer was not responsive
Hodgkin lymphoma [33]	40 M	3 doses of camrelizumab (200 mg every 2 weeks) subsequent to classic chemotherapy	Minimal change disease	Camrelizumab was discontinued and patient was treated with prednisone (1 mg/kg/day)	Renal function improved

Shown are the details of 13 cases which administration of anti-PD-1 antibody was followed by the appearance of glomerulopathy, either nephrotic syndrome or glomerulonephritis. Ipilimumab: monoclonal antibody targeting cytotoxic T-lymphocyte-associated protein 4 (CTLA-4); pembrolizumab, nivolumab: monoclonal antibody targeting PD-1; S-1: oral dihydropyrimidine dehydrogenase inhibitory fluoropyrimidine based on a biochemical modulation of 5-fluorouracil (5-FU)

The nephrotic syndromes associated with PD-1 immunotherapy include minimal change disease (MCD) and focal segmental glomerulosclerosis (FSGS). Both MCD and FSGS, as with other causes of nephrotic syndrome, manifest effacement of the glomerular filtration slits.

These are passageways between adjacent podocyte foot processes that provide size-selective and charge-selective regulation of the passage of plasma molecules from the glomerular capillary lumen, across the podocyte slit-diaphragm, into Bowman space and on into the proximal tubule lumen. Because the PD-1 immunotherapeutic response is associated with MCD and FSGS, factors involved in the development of these glomerulopathies may be important to the function of PD-1 receptors in the kidney.

MCD is an idiopathic nephrotic syndrome that is responsive to immunosuppressive therapies and rarely if ever progresses to end-stage kidney disease [39]. FSGS also involves podocyte injury and foot process effacement but unlike MCD, often progresses to end-stage kidney disease. FSGS is comprised of six syndromes [40].

- Primary FSGS*,* idiopathic, likely due to a circulating molecule
- Post-adaptive FSGS, due to a mismatch between glomerular load and glomerular capacity
- *APOL1* FSGS, due to susceptibility variants in the gene encoding apolipoprotein-L1, seen only in individuals with sub-Saharan ancestry
- High-penetrance genetic FSGS, associated with mutations in >50 nuclear and mitochondrial genes
- Virus-associated FSGS, associated with HIV-1, probably with cytomegalovirus and possibly with parvovirus B-19 and Epstein-Barr virus
- Medication-associated FSGS, due to androgens, bisphosphonates, interferon, lithium, chronic use of nephrotoxic drugs, and others

The functions of PD-1 molecules in HIV-1 [41], cytomegalovirus [42], and Epstein-Barr virus [43] have been reviewed elsewhere. PD-1 immunotherapies may therefore induce nephrotic syndromes by altering the activity of underlying identified genes or pathogens.

The nephritic syndromes most often occur in response to the generation of autoantibodies or to a dysregulated complement system. Autoantibodies against viral antigens or host tissue antigens can form circulating immune complexes, which can become passively trapped in the glomerular mesangium or subendothelial space within the glomerular capillary. Alternatively, autoantibodies or complement can directly bind components in the glomeruli [44]. Antibodies that bind complement factors can also alter complement activity. This may include autoantibodies directed against the complement C3 (C3NeF) or C4 (C4NeF) convertases. These nephritic autoantibodies are present in some forms of membranoproliferative glomerulonephritis (MPGN) [45]. The paracrine activity between complement factors and PD-1 molecules are reviewed elsewhere [46].

MPGN is a histopathologic pattern characterized by increased glomerular cellularity, capillary wall thickening and mesangial expansion. Patients with MPGN may present clinically with nephrotic syndrome or nephritic syndrome [47, 48]. Three different forms of MPGN are recognized. The first is immune complex-associated MPGN (IC-MPGN), which manifests in response to significant glomerular immunoglobulin deposition and activation of the complement classical pathway, leading to the formation of the complement membrane attack complex (MAC) on the surface of targeted cells [47, 48]. IC-MPGN is seen in lupus nephritis, immunoglobulin (Ig)-A nephropathy, infection-related glomerulonephritis and fibrillary glomerulonephritis with polyclonal immunoglobulin deposits [48]. The contribution of altered PD-1 activity in these diseases is discussed elsewhere [43, 49]. The additional forms are complement-mediated and identified as C3 glomerulopathy (C3G). The C3G category is divided into dense deposits disease (DDD) and C3 glomerulonephritis (C3GN). DDD involves osmiophilic electron-dense intramembranous deposits whereas C3GN includes nephritic factors and C3 deposits predominantly in the glomeruli, without intramembranous deposits [47, 48]. Crescentic glomerulonephritis can be identified in IC-MPGN and C3GN [50].

Pauci-immune glomerulonephritis (PIGN) is the most common cause of crescentic glomerulonephritis. Approximately 95% of PIGN cases exhibit antineutrophil cytoplasmic antibodies (ANCA) specific to myeloperoxidase (MPO-ANCA) or proteinase 3 (PR3-ANCA). Autoantibody binding to neutrophils induce neutrophil integrin expression and adherence to endothelial cells, which promotes transmigration across the endothelium [51]. These activated neutrophils release neutrophil extracellular traps (NETs) and granules that damage the endothelium. Ruptures in the glomerular basement membrane release plasma proteins and coagulation factors into Bowman space that promote parietal epithelial cell hyperplasia and form crescentic glomerulonephritis [50].

Tubulointerstitial inflammation and damage are common in progressive glomerular disorders and are the defining features in interstitial kidney disease [34, 35]. In a multicenter retrospective analysis, patients receiving PD-1/PD-L1 immunotherapy and a proton pump inhibitor or a CTLA-4 inhibitor developed tubulointerstitial nephritis in 93% of biopsied patients [37]. Renal biopsy is required for the diagnosis of anti-PD-1 immunotherapy-induced glomerulopathies and tubulopathies. In the USA, renal biopsy typically includes analysis of the tissue by light microscopy, electron microscopy and characterization of antibody deposition (IgG, IgM and IgA) and complement deposition. Immune cell infiltrates of myeloid lineage (monocytes, dendritic cells, neutrophils) and lymphoid lineage (T cells, B cells, plasma cells) may be identified by immunohistochemical staining, although this is typically a research procedure and not part of most clinical renal biopsy analyses [34, 35]. In a study assessing kidney biopsies obtained from anti-PD-1 immunotherapy patients that developed acute kidney injury, biopsy samples manifesting acute interstitial nephritis also showed increases in PD-L1 staining on tubular epithelial cell membranes compared to control biopsy samples with acute tubular necrosis [9]. Additional pathologies associated with glomerulonephritis as an adverse event of anti-PD-1 immunotherapeutic treatment are described in Table 1.

#### Macrophages in glomerulonephritis

**Fig. 2:**
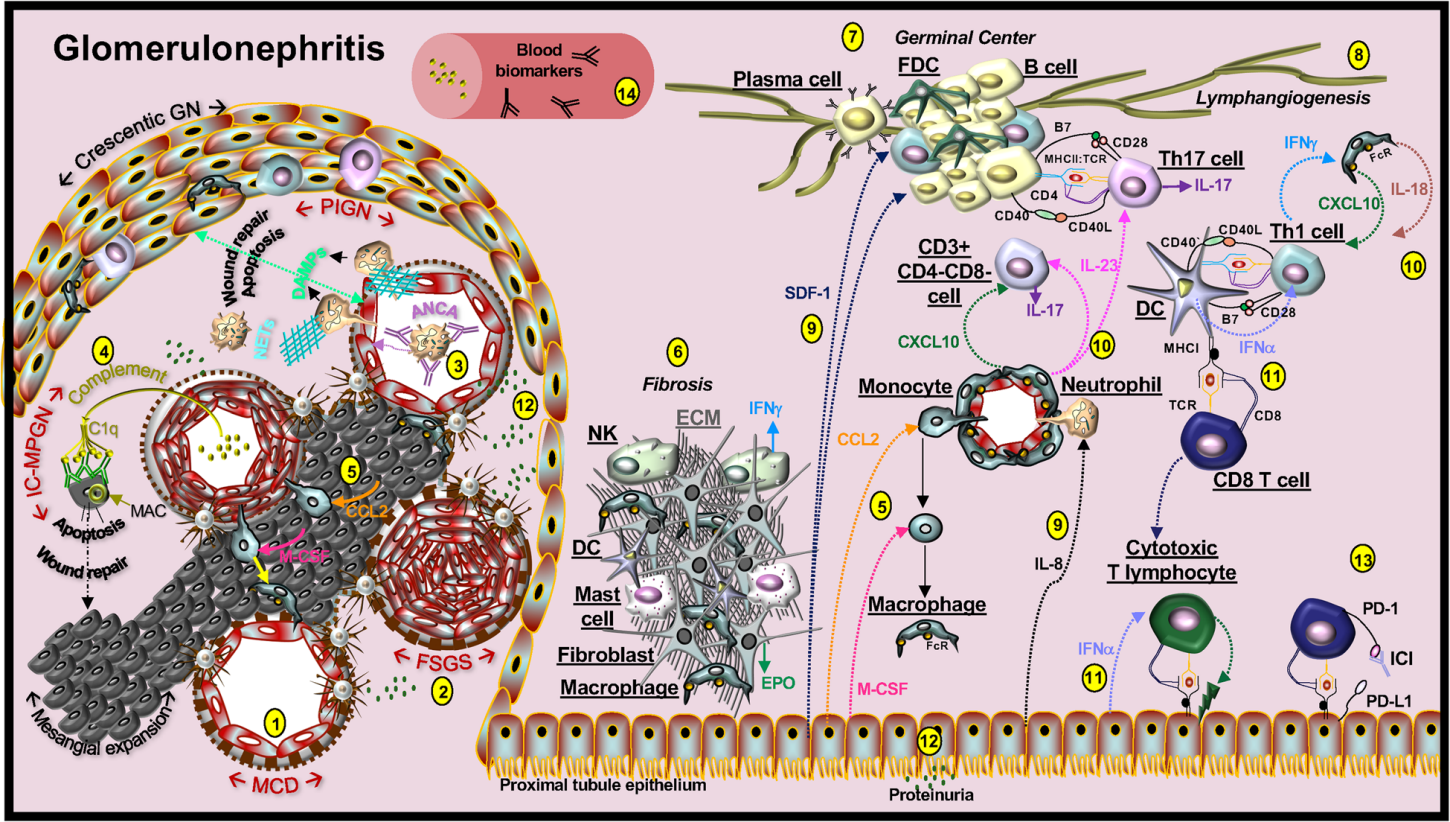
The microenvironment in glomerulonephritis. Types of glomerulopathy that may occur during PD-1 immunotherapy include minimal change disease (MCD), focal segmental glomerulosclerosis (FSGS), pauci-immune glomerulonephritis (PIGN), and membranoproliferative glomerulonephritis (MPGN). (1) In MCD, injured podocytes undergo foot process effacement whereby they lose filtration slits and cell-cell junctions, leading to loss of the size-selective and charge-selective filtration barrier. (2) FSGS also involves podocyte injury that progresses to the obliteration of the capillary lumens. (3) Antineutrophil cytoplasmic antibody (ANCA)-associated glomerulonephritis is a primary form of PIGN. Autoantibodies activate neutrophils to adhere to the endothelium, and these cells migrate into tissue and release damage-associated molecular pattern molecules (DAMPs). Activated neutrophils also release neutrophil extracellular traps (NETs) and granules that damage the endothelium and rupture the glomerular basement membrane. The subsequent release of plasma proteins and coagulation factors into Bowman space promote parietal epithelial cell hyperplasia and result in crescentic glomerulonephritis. (4) Immune complex MPGN (IC-MPGN) is characterized by capillary wall thickening, mesangial expansion, and may involve the formation of crescentic glomerulonephritis. Complement C1q binds autoantibodies which bind to mesangial cells and promote the deposition of the membrane attack complex (MAC). This pore-forming complex can damage cells in a lethal or sublethal manner. The subsequent wound repair response encourages cell proliferation. (5) Inflammatory cytokines induce mesangial cell and proximal tubule epithelial cell production of CCL2, which recruits macrophages and monocytes, and M-CSF, which promotes monocyte to macrophage differentiation. (6) Tubulointerstitial fibrotic lesions are characterized by excessive accumulation of extracellular matrix (ECM) molecules and the recruitment of natural killer (NK) cells, macrophages, dendritic cells (DCs), and mast cells. (7) Germinal center structures may form and contain follicular dendritic cell (FDC) networks, B and T cell aggregates and plasma cells can form. (8) Lymphangiogenesis can occur in glomerulonephritis. (9) Epithelial cells produce stromal cell-derived factor 1 (SDF1) to recruit B and T cells and IL-8 for neutrophil chemotaxis. (10) Myeloid cells produce IL-23 and IL-18, which induces the formation of Th17 and Th1 cells. These cells are are recruited to the interstitium and glomerulus, in part, by myeloid CXCXL10. NK cells and Th1 cells produce interferon (IFN)-γ. Most IL-17 producing cells in glomerulonephritis are CD3^+^CD4^-^CD8^-^ double negative. CD4+ IL-17 producing cells are also present. (11) IFN-α is primarily secreted by activated plasmacytoid DCs and by human proximal tubular epithelial cells in the interstitium. Cytotoxic T lymphocytes (CTL) are generated that destroy target cells. (12) Proteinuria is an identified marker of nephritis. (13) Immune checkpoint inhibitor (ICI) therapies may block homeostatic interactions that normally suppress inflammation. (14) Glomerulonephritis blood markers can include elevated levels of autoantibodies and altered complement turnover

Monocytes and macrophages are recruited by multiple factors, most notably by chemokine (C-C motif) ligand-2 (CCL2). This chemokine, also known as monocyte chemoattractant protein-1 (MCP-1), is produced in the kidney by mesangial cells and proximal tubule epithelial cells in response to cytokines (*e.g.,* tumor necrosis factor (TNF), interferon-γ, IL-1β) and by pathogen-associated molecular patterns (PAMPs) (*e.g.* lipopolysaccharide) [52, 53]. Mesangial cells and proximal tubule epithelial cells also produce macrophage colony-stimulating factor (M-CSF), which promotes differentiation of monocytes into macrophages [54] (Fig. 2). The pathogenic role of macrophages in glomerulonephritis has been established in animal models that demonstrate improved kidney function after macrophage depletion [55, 56]. In addition, in a glomerulonephritis murine model, glomerular macrophages expressed high levels of PD-L1 and proteinuria was suppressed either by blocking macrophage recruitment with anti-CD11b therapy or alternatively by antagonizing PD-L1 function with anti-PD-L1 therapy [55], suggesting a functional role of PD-L1 activated macrophages in the disease.

#### Fibrotic lesions and germinal centers in glomerulonephritis

In the setting of glomerulonephritis, interferon-γ-producing CD56^bright^NKp46^+^CD117^+^ natural killer (NK) cells are recruited in human fibrotic kidney tissue [57]. Interferon-γ produced by these cells induces production of the macrophage chemokine, CCL2, by mesangial cell and proximal tubule epithelial cells [53]. In response to tubular injury, macrophages, DCs, and mast cells are recruited and may contribute to fibrosis through production of transforming growth factor-β- or galectin-3-induced fibroblast proliferation and protease remodeling of the extracellular matrix (*e.g*., matrix metalloprotease-9, chymase, and tryptase) [14, 58].

The resulting tubulointerstitial fibrotic lesions manifest the following features: dysregulated production and organization of extracellular matrix proteins and reduced production of erythropoietin by fibroblasts [59], lymphatic growth [60] and the development of germinal center structures containing follicular dendritic cell (FDC) networks, B and T cell aggregates and plasma cells [61] (Fig. 2). In murine splenic germinal center formation, PD-1 and the ligands regulate the activity of B and T cells [62, 63], suggesting that germinal centers formed in the kidney may also require PD-1 cell signals, although this has not been shown. Erythropoietin therapy in the murine nephritis MRL/lpr strain promotes the formation of immunosuppressive T cell subsets [64]. Interstitial fibroblasts produce erythropoietin and with further investigation, could regulate PD-1 cell signals associated with T cell suppression. The PD-1 axis is therefore an attractive therapeutic target to suppress both germinal center activity and fibrosis in progressive kidney disease.

#### Neutrophils in glomerulonephritis

Although peripheral numbers of PD-L1-expressing neutrophils are elevated in lupus patients [65], this marker is generally absent from neutrophils in the human kidney and is absent from intraglomerular neutrophils in the NZM2328 murine model of lupus nephritis [55]. Neutrophils, which contribute to the pathology of inflammation in the glomeruli and the interstitium in lupus, are mainly recruited into the kidney by IL-8 released from cells activated by cytokines (*e.g.,* TNF or IL-1α), including podocytes, mesangial cells and proximal tubular epithelial cells [66, 67]. In lupus nephritis, enhanced production of IL-8 occurs in response to the cytokine IL-17 [68], which is produced predominantly by double-negative TCRαβ^+^CD3^+^CD4^-^CD8^-^ T cells [69]. The functions of double-negative T cells (*i.e.* those lacking CD4 and CD8), which can also express PD-1 [70], may therefore include the recruitment of neutrophils.

#### Lymphocytes in glomerulonephritis

Double-negative T cells [69], IL-17 producing CD4^+^ T cells (Th17) [71], and interferon-γ producing CD4^+^ T cells (Th1) [72] are recruited to the glomeruli and the interstitium. The chemokine receptor, CXCR3, is expressed on T cells and contributes to T cell recruitment in murine glomerulonephritis [73, 74]. In renal biopsy tissue from lupus nephritis patients, CXCR3^+^ T cells are present in the kidney and co-localize with cells which produce the CXCR3 ligand, CXCL10 [75]. Serum levels of CXCL10 in lupus patients are positively correlated with anti-DNA antibody levels [76]. Moreover, in a murine tumor model, anti-PD-1 therapy increases expression of CXCL10 at the tumor site [77]. Therapeutically targeting CXCL10 or CXCR3 may therefore reduce the inflammatory response in glomerulonephritis [75, 76] and may possibly affect the PD-1 axis. In addition, murine splenic self-reactive double-negative T cells can express PD-1 and this subset produces higher levels of IL-17 compared to their PD-1^-/-^ counterparts [70]. Double-negative T cells and FOX3P^+^ T regulatory cells (Tregs) are present in lupus renal biopsies [78]. The evaluation of PD-1 on these subsets in human kidney biopsy, as a biomarker or therapeutic target, remains to be explored.

#### Cytokines in glomerulonephritis

In glomerulonephritis, increased production of the cytokines IL-23 and IL-18, generated by antigen presenting cells, induces the expansion of IL-17-producing cells and interferon-γ-producing cells, respectively [79, 80]. In CD4^+^ Th17 and CD4^+^ Th1 cells, PD-1 expression is repressed [43]. Blocking IL-23 or IL-18 cell signals may therefore offer an approach to reduce inflammation and increase PD-1 expression in glomerulonephritis, which could be tested in relevant animal models.

#### Treatment of glomerulonephritis

Ideally, treatment of glomerulonephritis involves the direct targeting of the mechanisms underlying the disease process. Idiopathic glomerulonephritis and syndromes involving autoantibodies or complement commonly require immunosuppressive therapies and when these syndromes arise in the context of PD-1 therapies, withdrawal of immune-activating medications [34] (Table 2). Therapeutic algorithms support the use of glucocorticoids, calcineurin inhibitors, mycophenolate mofetil, azathioprine, anti-CD20 antibodies and most recently, belimumab, which targets the cytokine B-cell activating factor (BAFF) [86]. Anti-CD20 antibodies bind CD20 on B cells and plasma cells and initiate cell death through several mechanisms, including apoptosis, antibody-dependent cell-mediated cytotoxicity and complement-dependent cell lysis [87]. Indirectly, anti-CD20 antibodies may also protect the functions of podocytes through binding interactions with the cell surface receptor, sphingomyelin phosphodiesterase, acid-like 3b [88]. Anifrolumab, targeting the type I interferon receptor, has shown promise and may be soon approved for systemic lupus. Type I interferon cell signals also induce the expression of PD-L1 [89]. Additional research into the PD-1 response to pathogens and to autoimmunity may offer insight into the pathogenesis of glomerulonephritis and the adverse events that occur with PD-1 therapies.

**Table 2:** Identification PD-1, PD-L1 and PD-L2 expressing cells in the kidney

Cell	Marker	Human	Mouse
**Dendritic cells** in the interstitium and renal draining lymph [15]	PD-L1, PD-L2		Fluorescently labeled dextrans or OT-I cells injected into intravenously injected into C57BL/6, OT-I.RAG^−/−^, Thy1.1, and/or Rag^−/−^ mice
Human primary renal proximal tubular epithelial cells (**TECs**) [6]	PD-L1, PD-L2	Primary cultures of human TECs generated from healthy parts of tumor nephrectomies.	
Immunotherapy patient **TECs** [9]	PD-L1	Kidney biopsies from anti-PD-1 immunotherapy patients that developed acute interstitial nephritis exhibit elevated TEC PD-L1 staining compared to those with acute tubular necrosis	
Glomerulonephritis **macrophages** [55]	PD-L1		The lupus-prone NZM mouse strain +/- anti-glomerular basement membrane (GBM) antibodies
Clear cell RCC **macrophages** [81]	PD-L1, PD-L2	A mass cytometry-based atlas of 73 RCC tumor samples compared to five normal kidney controls	
**Unknown cell source** in clear cell RCC patients [82]	Soluble PD-L1	Sera soluble PD-L1 levels from 172 RCC patients correlates with pathologic features and patient outcome	
Clear cell RCC and non-clear cell RCC **tumors** [83]	PD-L1, PD-L2	In 425 resected RCCs, PD-L1 and PD-L2 expression is variable among histologic subtypes and associated with adverse outcomes in ccRCC	
CD4^+^CD25^hi^FOXP3^+^ (**Tregs**) in RCC patients [84]	PD-1	Primary tumor Tregs in 42 RCC patients displayed elevated PD-1 compared to cells in the peripheral blood of RCC patients and 15 healthy donors	
**CD8**^**+**^ **T cells** in RCC [85]	PD-1	*In situ* immunofluorescence spectral imaging of RCC tissue from nephrectomy revealed co-expression of PD-1 and TIM-3 on CD8^**+**^ associates with a more aggressive phenotype	

Shown are details of PD1, PD-L1 and PD-L1 identification in renal tumors and kidney cells from humans and mice, as described in nine published reports

#### Glomerulonephritis summary

The expression of PD-1 ligands on cells in the glomeruli or tubular epithelium during glomerulopathies has not been sufficiently explored. Macrophages and neutrophils are recruited during glomerulonephritis but only macrophages are identified to express PD-L1 in tissue. The expression and function of PD-1 on lymphocytes requires further investigation, particularly with respect to the double-negative T cells prominent in the kidney. Blocking CXCL10, IL-23 or IL-18 cell signals may induce lymphocyte PD-1 expression. Additional study of the PD-1 axis in glomerulonephritis may offer insight into the formation of germinal centers, fibrosis, and changes in erythropoietin production in the disease. Each of these concepts is important to understanding glomerulopathies that occur as an adverse event following to PD-1 immunotherapy.

### The PD-1 axis in RCC

Renal cell carcinomas are heterogeneous in histology, cell of origin, and driver mutations [90]. RCC develops from renal tubular epithelial cells in the proximal, distal and collecting tubules [91]. Similar to glomerulonephritis, RCC tumors typically exhibit a mix of myeloid and lymphoid cell infiltrates [81], fibrosis [92], and in aggressive forms, lymphangiogenesis [93]. Unlike glomerulonephritis, erythropoietin production may be elevated in RCC [94] and B cells are not commonly detected in RCC [95]. These latter inverse manifestations between the diseases may be associated with the differential expression of hypoxic cell signals that induce the production of erythropoietin [94] and promote B cell apoptosis [96].

#### RCC myeloid recruitment

**Fig. 3:**
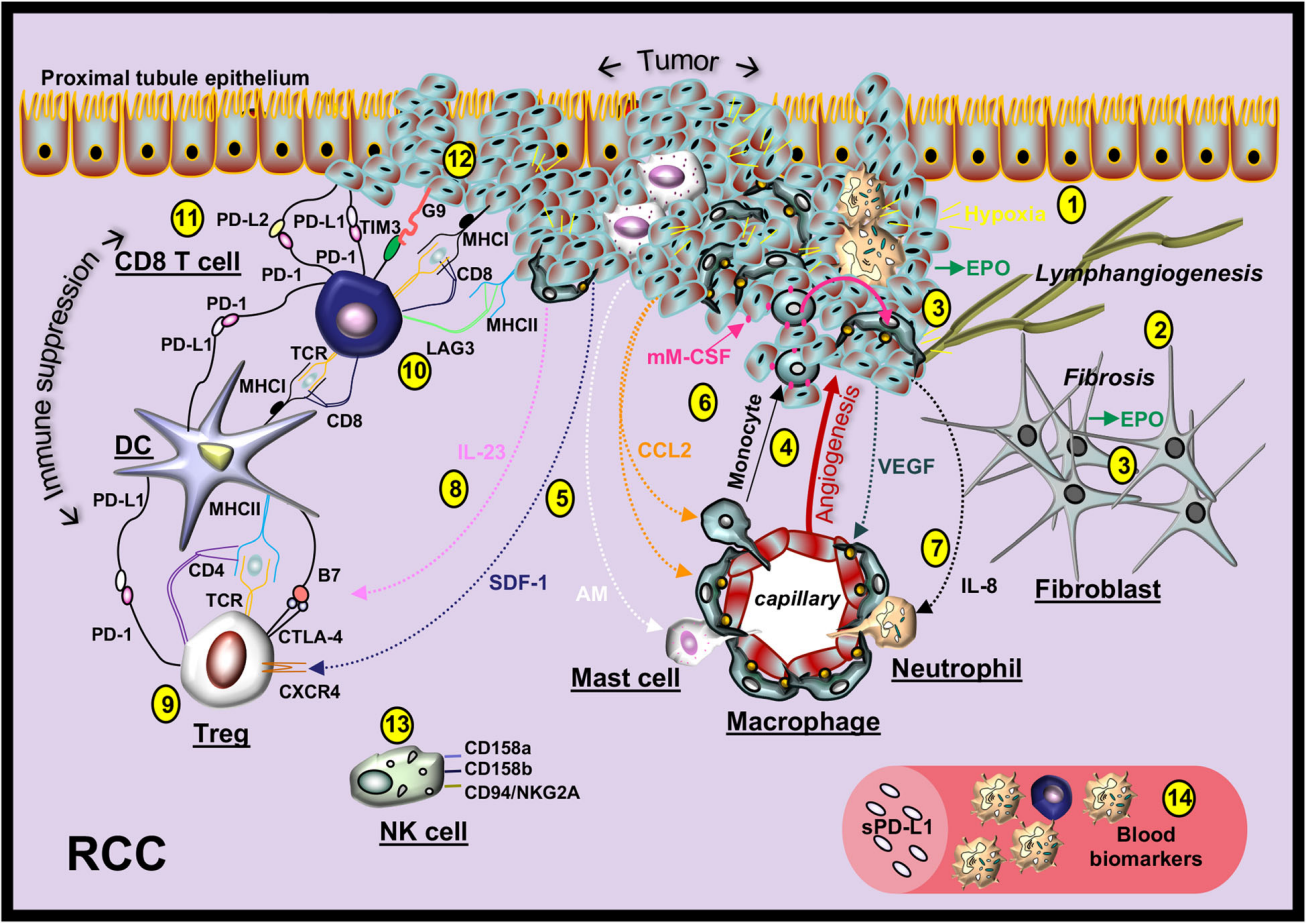
The microenvironment in renal cell carcinoma (RCC). (1) A hypoxic microenvironment is induced by tumor cells and recruited mast cells, macrophages and neutrophils. (2) Intratumoral fibrotic lesions and lymphangiogenesis can occur in RCC. (3) Erythropoietin (EPO) is produced by tumor cells in addition to interstitial fibroblasts. (4) Tumor cells and immune cells produce vascular endothelial growth factor (VEGF), which promotes angiogenesis. (5) Tumor cells and epithelial cells produce stromal cell-derived factor 1 (SDF1) and adrenomedullin (AM) involved in the recruitment of lymphocytes and mast cells, respectively. (6) Tumor cells produce CCL2, which recruits macrophages and monocytes and membrane-type M-CSF (mM-CSF), which promotes monocyte to macrophage differentiation. (7) Tumor cells produce IL-8, which recruits neutrophils. (8) Macrophages produce IL-23 involved in Treg function. (9) RCC Tregs express PD-1 and CTLA-4. (10) RCC CD8^+^ T cells express lymphocyte activating-3 (LAG3), which binds RCC MHC class II in promoting tolerance. (11) Tumor cells can express PD-L1 and PD-L2 and either can bind T cell PD-1 receptors. (12) Tumor cells can also express galectin-9 (G9) that binds to the suppressive CD8 T cell marker T-cell immunoglobulin and mucin-domain containing-3 (*TIM3*). (13) Immunosuppressive NK cells are recruited in RCC. (14) RCC peripheral blood markers can include an elevated neutrophil to lymphocyte ratio and elevated plasma levels of sPD-L1

In clear cell RCC, mutations in the von Hippel-Lindau (VHL) E3 ubiquitin protein ligase prevent VHL-induced ubiquitination of hypoxia-inducible factor (HIF)-1α and HIF-2α, which targets HIFs to the proteasome for degradation. This decreased degradation of HIF-1α and HIF-2α promotes hypoxic cell signals, which in turn promotes the production chemokines [97, 98]. RCC production of adrenomedullin [99], IL-8 [97], and CCL2 [100] contribute to the recruitment of mast cells, neutrophils and macrophages. The expression of membrane-bound macrophage colony-stimulating factor (mM-CSF) on RCC cells also contributes to the differentiation of monocytes to macrophages [101], which variably express PD-L1 and PD-L2 in tumor tissue [81]. The presence of these innate immune cells in RCC tumors enhances the production of vascular endothelial growth factor (VEGF), which is an angiogenic factor that has the potential to promote tumor growth and invasiveness [99, 101] (Fig. 3).

#### RCC myeloid function

In peripheral blood from RCC patients, an elevated neutrophil-to-lymphocyte ratio is associated with a poor prognosis. In a retrospective analysis of RCC patients treated with anti-PD-1 or anti-PD-L1-based regimens, a higher neutrophil-to-lymphocyte ratio measured six-weeks after therapy was independently associated with worse outcomes [102]. Because soluble PD-L1 is also a biomarker indicating poor prognosis in RCC [82], investigation of a mechanistic relationship between neutrophil function and soluble PD-L1 production may be warranted. Circulating neutrophil PD-L1 expression has not been evaluated in RCC. However, PD-1 is expressed on subsets of RCC-patient peripheral blood neutrophils, lymphocytes, and CD14^bright^ myeloid cells and the levels of PD-1 on these cells correlates positively with RCC disease stage [103]. This may indicate that PD-L1^+^ RCC tumors [104] or PD-L1^+^PD-L2^+^ RCC tumors [83] promote immunosuppression, in part by directly activating PD-1 on innate myeloid and lymphoid immune cells. Further studies are needed to test this hypothesis.

#### RCC T cell recruitment

Expression of the chemokine stromal cell derived factor-1 (SDF-1, also known as CXCL12) and its receptor, CXCR4, are induced by VHL inactivation and HIF stabilization. SDF-1 and CXCR4 are expressed in RCC tumors and are markers of poor prognosis [105]. Tregs isolated from primary RCC tumors express PD-1, CTLA-4 and high levels of CXCR4 [84]. NK cells can also express CXCR4 and PD-1 but are mostly characterized by the expression of immunosuppressive molecules CD158a, CD158b, and NKG2A/CD94 in RCC [106, 107]. The mechanisms involved in the recruitment of immunosuppressive NK cells and CD8 T cells in RCC are not well-understood.

#### RCC T cell function

CXCR4^+^ T cell lines treated with SDF-1 induce the production of VEGF [108]. This growth factor, which is also abundantly produced by RCC tumor cells, activates the expression of three CD8^+^ T cell checkpoint receptor genes, encoding lymphocyte activation gene-3 (LAG3), T-cell immunoglobulin mucin-domain containing-3 (TIM-3) and PD-1 [109]. In RCC tumor tissue, CD8^+^ T cells express these receptors [85, 110] (Fig. 3). Because CXCR4^+^ Tregs treated with a CXCR4 peptide antagonist effectively blocked Treg function and promoted interferon-γ production [84], CXCR4 is a potential therapeutic target in RCC. Interestingly, CXCR4 is also a target in lupus glomerulonephritis [111] and the levels of interferon-γ in this disease are elevated without the use of a CXCR4 antagonist [80]. Therefore, targeting VEGF receptors, PD-1, and/or additional checkpoints on T cells may be necessary to promote the development of tumor-specific T cells or effective T regulatory cells in glomerulonephritis.

#### RCC treatment

**Fig. 4:**
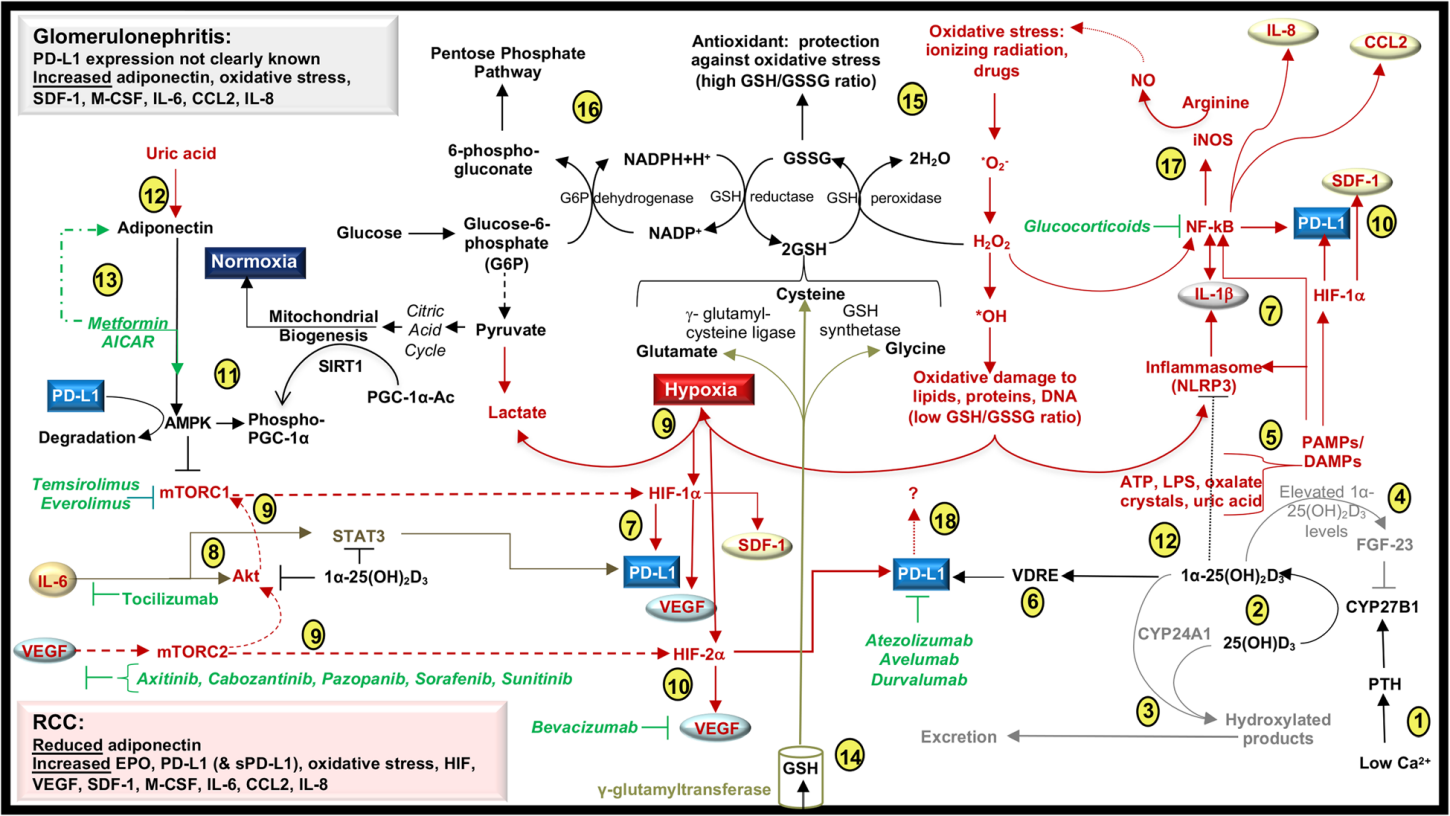
Possible proximal tubule epithelial cell signals in glomerulonephritis and renal cell carcinoma. (1) Low levels of calcium induce the release of parathyroid hormone (PTH) from parathyroid glands and this stimulates renal CYP27B1 expression. (2) 25(OH)D_3_, formed in the liver, is hydroxylated by CYP27B1 to form the active metabolite, 1α-25(OH)_2_D_3_. (3) CYP24A1 catalyzes the conversion of 25(OH)D_3_ and 1α-25(OH)_2_D_3_ into 24-hydroxylated products targeted for excretion. (4) Elevated levels of 1α-25(OH)_2_D_3_ induce the production of fibroblast growth factor-23 (FGF-23), which suppresses CYP27B1 transcription. (5) 1α-25(OH)_2_D_3_ may antagonize PAMP/DAMP-induced inflammasome activation. (6) Vitamin D response elements (VDRE) in the PD-L1 gene may be functionally active in the renal epithelium. (7) PAMPs/DAMPs, ROS, and IL-1β activate PD-L1 transcription factors, HIF-1α and NF-kB. PD-L1 expression can be blocked with monoclonal antibodies. (8) 1α-25(OH)_2_D_3_ antagonizes Akt and the transcription factor STAT3 that may be induced by IL-6 and mTORC2. IL-6 receptor monoclonal antibodies block IL-6 signals. (9) Hypoxia promotes the stability and activation of HIF-1α and HIF-2α. Both mTORC1 and mTORC2 are involved in HIF-1α regulation. mTORC2 regulates the expression of HIF-2α and is activated by VEGF cell signals. Tyrosine kinase inhibitors block VEGF receptor signals. (10) HIF-1α and HIF-2α induce the production of SDF-1 and VEGF. Antibodies that bind circulating VEGF block VEGF binding to its receptor. (11) Metformin-induced AMPK promotes PD-L1 phosphorylation and degradation. AMPK is also involved the phosphorylation of PGC-1α, which is additionally de-acetylated by sirtuins (SIRT1) during mitochondrial biogenesis and under conditions of normoxia. (12) Uric acid is a DAMP involved in the activation of the inflammasome and proximal tubule epithelial cell production of adiponectin. (13) Metformin and AICAR activate AMPK. Metformin therapy may induce the production of adiponectin. (14) γ-glutamyltransferase activity degrades glutathione (GSH) into its individual amino acids. (15) Reduced GSH scavenges free radicals and generates an oxidized form of GSH (GSSG). (16) GSH metabolism is associated with the activation of the pentose phosphate pathway. (17) NF-kB is antagonized by glucocorticoids and promotes the production of chemokines and ROS. (18) The cell signals that occur in response to PD-L1 ligation or blockade are not well characterized

RCC-induced inflammatory cytokines and VHL mutations induce cell signals that activate the mammalian target of rapamycin (mTOR) pathway. These signals also contribute to RCC tumor cell activation of HIF-1α and HIF-2α, which drive VEGF production and PD-L1 surface expression [112–114]. Treatments for RCC have primarily targeted VEGF ligands (bevacizumab), VEGF receptors (sorafenib, sunitinib, pazopanib, axitinib, cabozantinib), the mTOR pathway (temsirolimus, everolimus) and more recently PD-1 (pembrolizumab, nivolumab) and PD-L1 (atezolizumab, avelumab, durvalumab) [115]. The similar and yet distinct activation networks in these pathways are important in understanding and predicting therapeutic responses in monotherapies and combinatorial treatments and in selecting the right therapy for a particular patient, who may have co-morbidities that impinge on or intersect with these pathways (Fig. 4).

#### RCC summary

RCC tumor cells may express PD-L1 and/or PD-L2. Macrophages variably express PD-L1 and PD-L2 in tumor tissue. Circulating myeloid and lymphoid cells may express PD-1, which can be regulated by SDF-1 or VEGF cell signals. These pathways are activated by hypoxic cell signals that often include the dysregulation of mTOR. In the kidney, vitamin D3, 5’ AMP-activated protein kinase (AMPK) and glutathione are important to the physiological functions of the kidney and the cellular responses to hypoxia. The activation of PD-1 ligands by hypoxic cell signals also indicates that vitamin D3, AMPK and glutathione have a functional role in RCC and therapies that target mTOR, the PD-1 axis and VEGF.

### Vitamin D3 and the PD-1 axis

Vitamin D3, derived from ultraviolet-B radiation of 7-dehydrocholesterol in the skin or via dietary absorption, is 25-hydroxylated in the liver, predominantly by the P450 enzyme CYP2R1, to form 25(OH)D_3_. This molecule is subsequently 1-hydroxylated in the kidney by the proximal tubule epithelial cell enzyme 25-hydroxyvitamin D_3_ 1-α-hydroxylase (CYP27B1), forming the fully active metabolite, 1α-25(OH)_2_D_3_ [116, 117]. CYP27B1 activity is inhibited by fibroblast growth factor-23 (FGF-23), which in turn is induced by elevated levels of phosphate and 1α-25(OH)_2_D_3._ CYP27B1 activity is stimulated by parathyroid hormone (PTH). The synthesis and secretion of PTH is promoted by low plasma calcium and elevated plasma phosphate and is suppressed by physiologic plasma levels of 1α-25(OH)_2_D_3_ and fibroblast growth factor-23 [118]. With regard to catabolism, the mitochondrial 25-hydroxyvitamin D_3_ -24-hydroxylase enzyme CYP24A1 catalyzes the conversion of both 25(OH)D_3_ and 1α-25(OH)_2_D_3_ into 24-hydroxylated products targeted for excretion [119]. Through these cell signals, the tubular cell regulates the levels of the most potent form of vitamin D, 1α-25(OH)_2_D_3,_ and thereby maintains bone mineral homeostasis. Whereas calcium filtered at the glomerulus is reabsorbed along the nephron, phosphate is primarily reabsorbed in the proximal tubule.

The CYP27B1 and CYP24A1 enzymes are also expressed by nearly all immune cell subsets [120]. In chronic kidney disease patients, levels of serum phosphate and FGF-23 are elevated, possibly in response to CYP27B1 loss of function, resulting in increased CYP24A1 activity and vitamin D3 deficiency [121] (Fig. 4). Altered expression of the vitamin D receptor (VDR) is also a prognostic indicator in chronic kidney disease [3]. The downstream signals of 1α-25(OH)_2_D_3_ also promote and inhibit PD-1 ligand expression.

#### Vitamin D3 myeloid and epithelial cell signals

In human myeloid and skin epithelial cell lines, 1α-25(OH)_2_D_3_ enhances the expression of both PD-L1 and PD-L2 by binding to vitamin D response elements (VDRE) located near the gene [4]. Despite this similarity, additional cell signals in myeloid cells and epithelial cells manifest different responses to vitamin D. In myeloid cells, 1α-25(OH)_2_D_3_ interacts synergistically with pathogen- or damage-associated molecular patterns (PAMPs or DAMPs) in the activation of the NLRP3 inflammasome and subsequent production of IL-1β [122], which is a cytokine that induces PD-L1 expression [123]. 1α-25(OH)_2_D_3_ activation of the AKT/mTOR pathway also contributes to the formation of tolerogenic dendritic cells [124]. Regulation of the inflammasome by 1α-25(OH)_2_D_3_ in kidney epithelial cells has not been deeply explored. However, in human corneal epithelial cells, 1α-25(OH)_2_D_3_ antagonizes NLRP3 inflammasome activation and reduces the production of reactive oxygen species (ROS) and IL-1β [125]. 1α-25(OH)_2_D_3_ also antagonizes the activity of STAT3, AKT and mTOR in a human renal proximal tubular epithelial cell line (HRPTEpiC) [126]. Therefore, 1α-25(OH)_2_D_3_ may inhibit epithelial cell activation of the mTOR complexes, mTOR complex 1 (mTORC1) and mTORC2 [127], and also inhibit their downstream signals, HIF-1α and HIF-2α, which contribute to PD-L1 expression [114, 127]. Thus, the distinct cell signals that induce expression of PD-L1 on myeloid and epithelial cells may be regulated by 1α-25(OH)_2_D_3_ through the disparate expression of the VDR and/or additional cell signals from PAMPs/DAMPs and cytokines that modulate signaling downstream of the VDR.

#### Vitamin D3 T cell signals

VDR activation in T cells induces transcriptional repression of *IL-17A* but induction of *FOXP3* [128], which also contain VDREs [129]. These responses may offer a mechanism to explain the inverse correlation between low serum 25(OH)D_3_ levels and the elevated IL-17 levels in SLE patients with 25(OH)D_3_ deficiency [130]. Treg and Th17 cells rely upon mTORC1 activity during differentiation [131, 132]. This suggests that rapamycin analogs may antagonize the generation of both T cell subsets, which are differentially generated in response to 1α-25(OH)_2_D_3_ and differentially express PD-1 [43]. A possible mechanism to explain the 1α-25(OH)_2_D_3_ response in T cells may involve mTORC2 activity, which promotes the development of Tregs [133], possibly via VEGF signaling [134]. A downstream signal of mTORC2 is mTORC1, which is a primary target in current clinical trials of RCC [115] and lupus [135]. Further research into 1α-25(OH)_2_D_3_ regulation in immune and non-immune cells may yield insight into the functions of rapamycin analogs and other therapeutics that target products of mTOR activation (*e.g*., VEGF and PD-L1).

#### Vitamin D3 summary

Altered expression of the VDR is associated with chronic kidney disease and affects mTOR signals and the expression of PD-1 and its ligands. VDR-induced mTORC1/2 signals differ in immune cells compared to epithelial cells, which may offer insight into the efficacies of mTOR inhibitors and PD-1 immunotherapies. The link between mTOR pathways and hypoxic metabolism also suggests that vitamin D3 is involved in the regulation of cellular hypoxia, which is a metabolic pathway highly activated in RCC and known to induce the expression of PD-1 ligands.

### AMPK and the PD-1 axis

In immune cells and renal parenchymal cells, 5’ AMP-activated protein kinase (AMPK) is activated in response to low intracellular ATP levels, resulting in AMPK-mediated phosphorylation of multiple substrates involved in stimulating energy production and minimizing energy consumption [136]. AMPK promotes aerobic metabolism by activating a co-factor in mitochondrial biogenesis, specifically peroxisome proliferator -activated receptor gamma coactivator 1-alpha (PGC-1α). AMPK also antagonizes mTORC1 and NF-κB cell signals, which collectively promote hypoxia [137].

#### Adiponectin-induced AMPK

Adiponectin is an endogenous activator of AMPK and induces anti-inflammatory responses in innate immune cell subsets [138]. This protein hormone is predominantly secreted by adipocytes but can also be generated by proximal tubule epithelial cells in response to a DAMP, soluble uric acid [139]. Adiponectin exhibits structural homology with the complement component, C1q, and both of these molecules activate AMPK in murine bone marrow-derived macrophages [140]. In lupus nephritis patients, both urine [141] and serum [142] levels of adiponectin are elevated compared to controls. In contrast, lower plasma adiponectin levels are associated with an increased incidence of RCC [143] (Fig. 4). Because serum levels of adiponectin tend to decrease with obesity [144], obesity may be a factor in the progression of RCC. Moreover, the adenosine analogue, 5-amino-4-imidazole carboxamide riboside-1-β-D-ribofuranoside (AICAR), also activates AMPK and inhibits both mTORC1 activity and PD-L1 expression in lung cancer models [145], suggesting that adiponectin-induced AMPK activation could have comparable functions in the kidney. Possibly, higher levels of adiponectin in glomerulonephritis reduce PD-L1 expression whereas lower adiponectin levels in RCC increase PD-L1 expression on proximal tubule epithelial cells.

#### Metformin-induced AMPK

Metformin, a plant-derived biguanide, is commonly used in the treatment of diabetes mellitus and induces AMPK activation through mechanisms that have not been fully elucidated [146]. In cultured breast cancer cells, metformin-induced AMPK activity promotes PD-L1 phosphorylation and subsequent endoplasmic-reticulum-associated PD-L1 degradation [147]. In a murine melanoma model, a combination of metformin and PD-1 blockade results in improved intratumoral T-cell function and tumor clearance [148]. The effects of metformin on PD-L1-expressing proximal tubular epithelial cells and RCC tumor cells have not been fully explored. Metformin is being assessed in reducing disease activity flares in lupus patients (ClinicalTrials.gov Identifier: NCT02741960) and a combination of metformin and PD-1 blockade (nivolumab) is being tested in subjects with stage III-IV non-small cell lung cancers that cannot be surgically removed (ClinicalTrials.gov Identifier: NCT03048500). The outcomes of these trials may yield insight into the mechanisms of metformin in PD-1 immunity and kidney disease.

Moreover, retrospective studies of RCC patients receiving metformin for diabetes mellitus demonstrated improved overall survival in these patients compared to metformin non-users [149], particularly in localized non-metastatic RCC [150]. A retrospective study of type 2 diabetes patients showed that serum adiponectin levels increased with metformin therapy [151]. Higher levels of adiponectin with metformin therapy may therefore suggest a mechanism for improved outcomes in RCC [149] but possibly not in glomerulonephritis, where adiponectin levels are elevated in the absence of metformin [142]. However, because both metformin [152] and adiponectin [138] promote anti-inflammatory responses in immune cell subsets, additional study is warranted.

#### AMPK summary

AMPK activation in the kidney may inhibit mTORC1 cell signals and the expression of PD-L1. Adiponectin is an AMPK ligand that may be elevated in the plasma of lupus nephritis patients but reduced in RCC patients compared to controls. Obesity may therefore regulate PD-L1 expression in the kidney through adiponectin cells signals. The potential regulatory role of metformin in PD-L1 expression in the kidney requires further study.

### Glutathione and the PD-1 axis

*De novo* production of the reduced form of glutathione (GSH) occurs via ligase and synthetase reactions that form a tripeptide composed of glutamic acid, cysteine, and glycine [153]. While most cells synthesize GSH, the liver is the primary source of circulating GSH, and the kidney is the primary tissue involved in the uptake of GSH from blood [154]. Renal proximal tubule epithelial cell γ-glutamyltransferase activity degrades GSH into its constituent amino acids. The recycling of these amino acids back into GSH and the conversion of oxidized glutathione (GSSG) to GSH via a GSH reductase are components of the GSH salvage synthesis pathway [153]. GSH reductive capacity is primarily driven by the pentose phosphate pathway, which generates GSH. Oxidation of GSH occurs through reactions with hydroxyl radicals (^•^OH) or superoxide anion (O_2_^•−^). GSH can also be as a co-substrate of GSH peroxidases that reduce lipid peroxides and hydrogen peroxide (H_2_O_2_) into alcohol or H_2_O, respectively [153] (Fig. 4). Genetic variants in GSH enzymes (*e.g., GPX1, GPX3*) [155] or factors involved in the transcriptional regulation of GSH genes *(e.g., NFE2l2*, *KEAP1*) [156] may promote oxidative stress in both glomerulonephritis [157] and RCC [158]. These mutations may also affect plasma 25(OH)D_3_ levels since GSH plasma levels are positively correlated with plasma 25(OH)D_3_ levels in a study of obese adolescents, with further support from mouse studies [159]. A lack of GSH may also increase ROS, which in turn activates the transcription factors, NF-kB and HIF-1α, that regulate expression of *CD274*, encoding PD-L1 [160]. The role of ROS or GSH in the expression of PD-L1 in kidney immune or parenchymal cells remains to be fully explored.

#### Glutathione summary

By recycling GSH, kidney epithelial cells are constantly supplied with the factors required to synthesize this antioxidant. GSH can neutralize ROS involved in the activation of transcription factors that induce the expression of the PD-1 ligands. Changes in GSH plasma levels may be associated with PD-L1 expression and function.

### Comparing the immunobiology of RCC and glomerulonephritis

The expression of PD-1 molecules in the kidney as discussed and displayed (Table 2) reveals the importance of these checkpoints and need for the additional study of PD-1 and the PD-1 ligands in the kidney. The immunobiologic similarities and differences between glomerulonephritis and RCC may offer insight into the functions of these checkpoints. In both glomerulonephritis and RCC, epithelial cells and/or tumor cells produce CCL2 [53, 100], M-CSF [54, 101], and IL-8 [66, 97], which recruit monocytes and macrophages, promote monocyte-to-macrophage differentiation, and recruit neutrophils, respectively. RCC cells also produce the mast cell chemokine adrenomedullin [99], which could also be a factor in mast cell recruitment in glomerulonephritis [14, 161]. Notably, B cell recruitment and the formation of germinal centers in the kidney are seen, albeit rarely, in chronic glomerulonephritis [61] but not in RCC [95]. In the murine spleen, macrophages act as regulators of germinal center formation and this may indicate that the activation of macrophages is pivotal to germinal centers formation in the kidney [162]. Macrophages also produce IL-23 and IL-18, which contribute to the generation of Th17 and Th1 T cell subsets, respectively, in glomerulonephritis [79, 80]. In an orthotopic model of kidney cancer, IL-23 blockade improved survival and enhanced the efficacy of PD-1 blockade, possibly by inhibiting IL-23-induced Tregs [163]. In a melanoma model, IL-18 therapy augments PD-L1 blockade by activating CD8^+^ T cells and NK cells but suppressing Tregs [164]. This tends to support *in vitro* data involving IL-2/IL-18 activated-NK cell cytotoxic killing of RCC cell lines [165]. The functional responses of IL-23 and IL-18 on double negative TCRαβ^+^CD3^+^CD4^-^CD8^-^ T cells, which are highly expressed in the kidney [69] and can also express PD-1 [70], requires further study. Continued research into the functions of macrophages and the IL-18/IL-23 balance may offer insight to the PD-1 axis in kidney disease.

The chemokine SDF-1 and its receptor CXCR4 are expressed both in glomerulonephritis and RCC [105, 111]. Data indicating that SDF-1 ligation to CXCR4 induces IL-6 production [166] may provide a rationale for the targeting of IL-6 in both diseases [167, 168]. An additional chemokine receptor, CXCR7/ACKR3, recognizes SDF-1, adrenomedullin and CXCL11 [169, 170]. This latter chemokine is also a CXCR3 ligand and a member of the interferon-inducible chemokine family, which also includes CXCL9, CXCL10 and CXCL11 [169]. Each of these CXCR3 family chemokines can be dysregulated in both glomerulonephritis [171] and RCC [172]. To add to the complexity, variants of the gene encoding CXCR3, CXCR3A and CXCR3B, are involved in promoting and inhibiting endothelial cell growth upon ligand binding, respectively [173]. Because CXCR3 ligation promotes the anti-tumor effects of PD-1 blockade in mice [174], further study of the CXCR3 variants, their ligands, and competing receptors on immune and parenchymal cells in the kidney may yield novel insights.

In RCC, hypoxia promotes the production of VEGF [113] and erythropoietin [94]. The paracrine signals between VEGF and HIF pathways may promote PD-L1 expression in RCC (Fig. 3) and lend support to clinical trials involving VEGF inhibitors and PD-1 targeted therapies [175]. Because VEGF is not only an important factor in angiogenesis, but also enhances the function of Tregs [134], T cell VEGF receptors may be a therapeutic target in glomerulonephritis and RCC. Targeting erythropoietin receptors on T cells may also be warranted, as erythropoietin is an additional factor that promotes the formation of immunosuppressive T cell subsets [64]. Consequently, erythropoietin and VEGF are candidate factors in regulating the expression and function of PD-1 in T cells but this remains to be demonstrated.

Finally, PD-L1 gene transcriptional activators include STAT1/3, NF-κB, HIF-1α, and HIF-2α [176]. These transcription factors are induced by cytokine (*e.g.,* IL-6, interferons, IL-1β) and/or hypoxic cell signals (Fig. 3). These cell signals are differentially modulated by 1α-25(OH)_2_D_3_ in immune cells [122, 124] and epithelial cells [125, 126]. A mechanism underlying these diverse responses may involve GSH, particularly since renal proximal tubule epithelial cells are the primary cells responsible for GSH uptake from plasma [154]. GSH contributes to regulating the production of degradative enzymes (CYP24A1) and anabolic enzymes (CYP27B1), both of which determine 1α-25(OH)_2_D_3_ plasma and tissue levels [177]. In cultured proximal tubule epithelial cells, 1α-25(OH)_2_D_3_ also antagonizes mTOR [126], which is a direct target of rapamycin analogs and an indirect target of metformin and adiponectin (Fig. 3). Future research into the crosstalk among these pathways may identify markers for resistance to PD-1 therapy and additional therapeutic targets in glomerular disease.

## Conclusion

Blocking PD-1 ligation with anti-PD-1 immunotherapy agents can induce various forms of glomerulonephritis. To understand the etiology of the effect, we have reviewed the possible expression and function of PD-1 receptors in a healthy kidney, glomerulonephritis and RCC. The PD-1 ligands, PD-L1 and PD-L2, are present on healthy proximal tubule epithelial cells *in vivo* and their expression is increased in some forms of RCC. Increased expression of PD-L1 also occurs in anti-PD-1 immunotherapy-induced acute interstitial nephritis, suggesting that the response may also occur in glomerulonephritis. In RCC, an elevated plasma level of soluble PD-L1 is a poor prognostic indicator. The cellular source and function of soluble PD-L1 in RCC has yet to be fully evaluated. In glomerulonephritis, serum levels of soluble PD-L1 could also be examined as a possible biomarker, particularly since 1, 25 dihydroxy-vitamin D3 is a factor in PD-L1 expression and levels of this vitamin are often low in chronic kidney disease in the absence of adequate supplementation. Mast cells, macrophages, neutrophils and T cells are similarly recruited to the kidney in both diseases, in which immunological processes manifest very differently. Because CXCR3 activation has a role in promoting the anti-tumor response in PD-1 immunotherapy, a better understanding of the ligands that bind CXCR3 variants (CXCR3A, CXCR3B) and competing receptors (CXCR4, CXCR7) is needed. Myeloid cells tend to express PD-L1 in glomerulonephritis but may express PD-1 in RCC. The cell signals from these receptors on innate immune cells in the context of disease and immunotherapy requires further study. Erythropoietin, vitamin D3 and VEGF promote the formation of Tregs, suggesting that receptors for these molecules on T cells might be therapeutic targets in kidney disease and might also contribute to a possible PD-1 immunotherapeutic response. Because hypoxic cell signals induce the expression of PD-L1 molecules and promote B cell apoptosis, endogenous molecules that regulate hypoxic cell signals (*e.g*., AMPK, vitamin D3, GSH) and drugs that block hypoxic responses (*e.g*., rapamycin analogs, glucocorticoids, VEGF inhibitors) may increase the efficacy of PD-1 immunotherapies and the functions of B cells, including germinal center formation. Understanding these interconnected networks that regulate the PD-1 response will be particularly important to identifying patients at increased risk for the development of glomerulonephritis as a complication of PD-1 immunotherapy.

## Data Availability

All data generated or analyzed during this study are included in this published article

## Abbreviations

AICAR: 5-amino-4-imidazolecarboxamide riboside-1-β-D-ribofuranoside
AMPK: AMP-activated protein kinase
CCL2: Chemokine (C-C motif) ligand-2
CTLA-4: Cytotoxic T-lymphocyte-associated protein 4
DAMPS: Damage-associated molecular patterns
DC: Dendritic cell
FcR: Fc receptor
FDC: Follicular dendritic cell
FGF-23: Fibroblast growth factor-23
G9: Galectin-9
GSH: Reduced glutathione
GSSG: Oxidized glutathione
IL: Interleukin
HIF: Hypoxia-inducible factor
INF: Interferon
LAG3: Lymphocyte activation gene-3
MAC: Membrane attack complex
MHC: Major histocompatibility complex
M-CSF: Macrophage colony-stimulating factor
mM-CSF: Membrane M-CSF
mTOR: Mammalian target of rapamycin
mTORC: mTOR complex
NK: Natural killer
PAMPs: Pathogen-associated molecular patterns
PD-1: Programmed death receptor-1
PD-L1: PD-1 ligand
PD-L2: PD-1 ligand
PTH: Parathyroid hormone
RCC: Renal cell carcinoma
ROS: Reactive oxygen species
SDF-1: Stromal cell-derived factor-1
SIRT: Sirtuin
sPD-L1: Soluble PD-L1
TCR: T cell receptor
TIM-3: T-cell immunoglobulin mucin-domain containing-3
TNF: Tumor necrosis factor
VEGF: Vascular endothelial growth factor
VDR: Vitamin D receptor
VDRE: Vitamin D response elements
VHL: von Hippel-Lindau
